# Novel synthesis of holey reduced graphene oxide (HRGO) by microwave irradiation method for anode in lithium-ion batteries

**DOI:** 10.1038/srep29854

**Published:** 2016-07-26

**Authors:** Edreese Alsharaeh, Faheem Ahmed, Yazeed Aldawsari, Majdi Khasawneh, Hatem Abuhimd, Mohammad Alshahrani

**Affiliations:** 1 College of Science and General Studies, Alfaisal University, P.O. Box 50927, Riyadh, 11533, Saudi Arabia; 2 National Nanotechnology Center, King Abdulaziz City for Science and Technology, P.O Box 6086, Riyadh 11442, Saudi Arabia

## Abstract

In this work, holey reduced graphene oxide (HRGO) was synthesized by the deposition of silver (Ag) nanoparticles onto the reduced graphene oxide (RGO) sheets followed by nitric acid treatment to remove Ag nanoparticles by microwave irradiation to form a porous structure. The HRGO were characterized by X-ray diffraction (XRD), field-emission scanning electron microscopy (FESEM), transmission electron microscopy (TEM), ultra violet-visible spectroscopy (UV-Vis), thermogravimetric analysis (TGA), and Raman spectroscopy. These novel HRGO exhibited high rate capability with excellent cycling stability as an anode material for lithium-ion batteries. The results have shown an excellent electrochemical response in terms of charge/discharge capacity (423 mAh/g at 100 mA/g). The cyclic performance was also exceptional as a high reversible capacity (400 mAh/g at 100 mA/g) was retained for 100 charge/discharge cycles. This fascinating electrochemical performance can be ascribed to their specific porous structure (2–5 nm pores) and high surface area (457 m^2^/g), providing numerous active sites for Li^+^ insertion, high electrical conductivity, low charge-transfer resistance across the electrolyte–electrode interface, and improved structural stability against the local volume change during Li^+^ insertion–extraction. Such electrodes are envisioned to be mass scalable with relatively simple and low-cost fabrication procedures, thereby providing a clear pathway toward commercialization.

Li-ion battery (LIB) has shown a great potential to fulfil the growing energy demands for hybrid electric vehicle, smart grid applications, and portable electronic devices, due to its advantages such as high power and energy density, long cycle life and structure stability^1^,^2^,^3^,^4^. The energy density and performance of LIBs are largely determined by the chemical and physical properties of the cathode and anode materials^5^,^6^,^7^. Although significant progress has been made in improving the properties of electrode materials over the years^8^,^9^,^10^, there is immense interest to develop new electrode materials that can boost both the energy and power densities of LIB^11^,^12^,^13^.

As an allotrope of carbon, graphene consists of a single planar sheet of sp^2^-bonded carbon atoms that are densely packed in a honeycomb crystal lattice. Graphene has been demonstrated to be a promising anode material for LIBs due to its intriguing physical and chemical properties, such as high electrical conductivity, large specific surface area, and mechanical robustness^14^,^15^,^16^. However, due to the large surface area and strong van der Waals interaction between sheets, the graphene nanosheets are prone to aggregation after drying to form a paper-like structure^12^,^17^. As a result, although the in-plane diffusivity of Li^+^ions in the graphene stack is high, their cross-plane diffusivity is low, and the migration of Li^+^ions into and out of the graphene anode may occur predominantly at the edge of the stack. This greatly limits the electrochemical performance, particularly at high C rates^12^,^13^,^14^,^15^,^18^,^19^,^20^.

In a quest to overcome the aforementioned limitations of graphene, the focus of current research is to improve the electrochemical performance of graphene by creating holes into the planar sheet, since the holes can provide a high density of crossplane diffusion channels for Li^+^ions^21^. In particular, the nanopores in HRGO sheets are large enough to function as the ion diffusion shortcuts between different layers of graphene to greatly speed up the ion transport across the entire film and facilitate ion access to the entire surface area, which is not possible with non-holey graphene^21^,^22^. Together, these combined features make it possible for HRGO to achieve high volumetric capacitance while retaining high gravimetric capacitance and excellent rate capability.

For the bulk preparation of graphene, a standard method starts with strong oxidation of natural graphite into graphene oxide (GO) that is dispersible in aqueous solutions as exfoliated monolayer or few-layered sheets^23^. The exfoliated GO sheets may then be chemically or thermally converted into graphene–or more accurately “reduced graphene oxide” (RGO). Recently, there have been a few reports on novel types of graphene structures that feature large pore openings (i.e., holes) on the conjugated carbon surface^24^,^25^,^26^,^27^,^28^. Compared to conventional defects that often take extensive efforts to observe using high-resolution microscopic techniques^29^, the pore openings in these novel holey graphene (HRGO) structures are much larger (ranging from a few to hundreds of nanometres) and are easily identified.

In this work, we report a conceptually different approach to enhance the power capability of graphene-based electrodes, by introducing holes into graphene sheets using Ag nanoparticles by a facile microwave irradiation method. To the best of our knowledge, this is the first demonstration of microwave irradiated holey graphene (HRGO) electrodes in LIB. The results have shown an excellent electrochemical response in terms of charge/discharge capacity, rate capability, and cyclic performance. This method can be extended to rational design of HRGO and its hybrids with new functionalities.

## Experimental Details

### Materials

Extra pure graphite powder (>99.5%) was purchased from Merck, and hydrazine hydrate (HH, 80%) was obtained from Loba Chemi. Pvt. Ltd. Silver nitrate (AgNO_3_), potassium permanganate (KMNO_4_, >99%), and hydrogen peroxide (H_2_O_2_, 30%) were obtained from Merck. Other solvents and chemicals were of analytical grade and used without further purification.

### Preparation of AgNPs/RGO

The dried GO^30^ (500 mg) was stirred and sonicated in 50 ml of deionized water until a homogeneous yellow dispersion was obtained. The GO can be dispersed easily in water due to the presence of a variety of hydrophilic oxygen groups (OH, O and COOH) on the basal planes and edges. Now, add 200 μL of 20% AgNO_3_ followed by sonication for a minute and then stirring in which 12 μL of Hydrazine Hydrate was added drop wise while stirring. The solution was placed inside a conventional microwave. The microwave oven (Kenwood MW740) was operated at full power (900 W) for a total reaction time of 2 min. The yellow dispersion of GO gradually changed to a black color indicating the completion of the chemical reduction to RGO. The RGO sheets decorated with Ag nanoparticles were separated using a centrifuge operated at 5000 rpm for 15 min and dried at 60 °C overnight. The product was then collected.

### Preparation of HRGO

**Figure 1:**
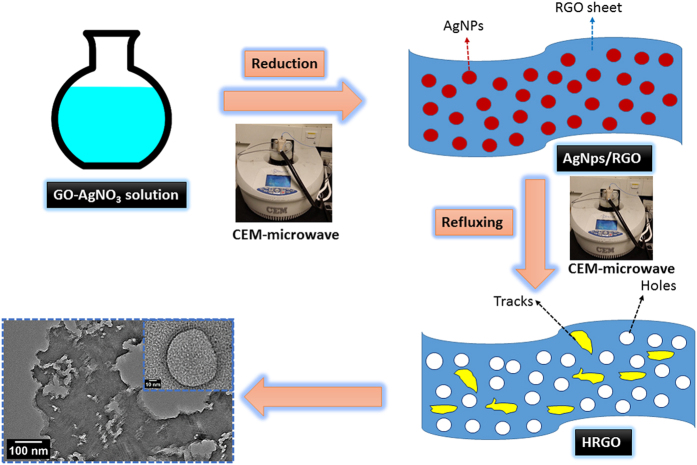
Schematic representation of the formation of HRGO by microwave irradiation.

In a typical reaction, Ag/RGO sample was heated in air using a furnace at a temperature of 300 °C for 2 h. Then, the furnace heated Ag/RGO sample (100 mg) was refluxed in diluted nitric acid (2.6 M, 50 mL) and put in microwave system for 30 min to remove Ag. Upon cooling, the slurry was centrifuged and the supernatant was discarded. The solid was then repeatedly washed with water in up to ten more redispersion–centrifugation cycles until the supernatant reached neutral (pH ~ 7). The solid was then dried either at 60 °C in vacuum to obtain the final HRGO product. The detailed schematic representation of the formation of HRGO is shown in Fig. 1.

### Electrochemical testing

The electrochemical performance was evaluated using two-electrode coin half-cell with a polypropylene membrane separator (Celgard 2325, Celgard, Inc., USA) as separator and Li-metal (purity 99.9%) as counter electrode. 1.2 M LiPF_6_ in EC/DMC (1:1, v/v) was used as an electrolyte. Cells were assembled in a dry room and galvanostatically charged/discharged at current densities of 100 mA in a potential range of 0.005 to 3 V, using a multi-channel battery tester (TOYO TOSCAT-3100U).

Cyclic voltammetery (CV) and Electrochemical impedance spectroscopy (EIS) analyses were carried out through an electrochemical working station (Gamry 3000). CV was performed at 5 mV/s scan rate in a voltage range of 0.005–3 V. While, EIS was performed by applying a perturbation voltage of 10 mV/s in a frequency range between 1 Hz and 100 kHz.

### Characterization

The Fourier transform infrared spectroscopy (FTIR; Thermo Scientific Nicolet-iS10, USA) spectra of the product were recorded in the range of 4000–400 cm^−1^. The X-ray diffraction (Philips-PW 1729, Holland) of the material were investigated with Cu radiation [30 kV, 40 mA, Ka radiation (1.54430 A°)]. The X-ray photoelectron spectroscopy (XPS) analyses were performed on an ESCALABMK II X-ray photoelectron spectrometer with Mg-Ka radiation. The Raman spectra were measured using a Bruker Equinox 55 FTIR spectrometer equipped with an FRA106/S FT-Raman module and a liquid N_2_-cooled Ge detector using the 1064 nm line of a Nd:YAG laser with an output laser power of 200 mW. A scanning electron microscope (SEM, FEI Quanta 200) was employed to study the morphology of the product after they were mounted on the nanocomposite slabs and coated with gold via sputtering system (Polaron E6100, Bio-Rad). High-resolution transmission electron microscopy (HRTEM) was performed using (JEOL JSM-2100 F, JEOL) and operated at 200 kV. A drop of the specimen dispersed in ethanol was placed on copper grids and dried for the studies. The thermogravimetric analyses (TGA) of the product were studied using a NETZCH 209 F1 thermogravimetric analyser. The surface area and pore diameter were analysed with a surface area analyser (ASAP 2020, Micromeritics) using physical adsorption–desorption of N_2_ at liquid-N_2_ temperature. The specific surface area and pore size distribution were calculated according to the Brunauer–Emmett–Teller (BET) method and the Barrett–Joyner–Halenda (BJH) method, respectively.

## Results and Discussion

**Figure 2:**
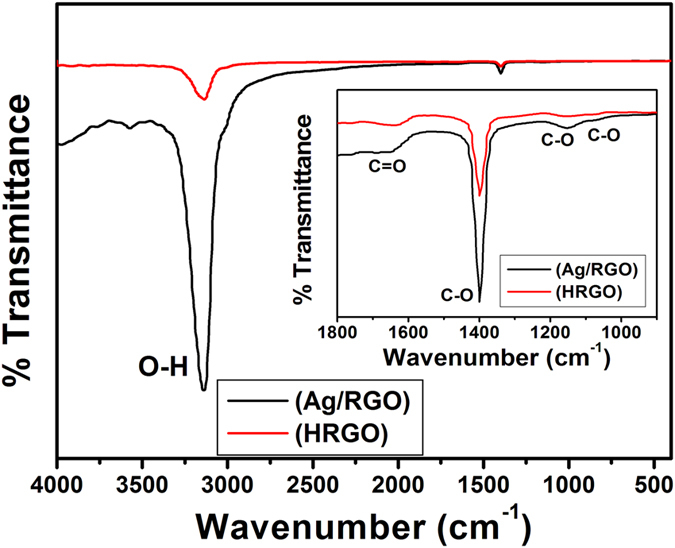
FTIR spectra of Ag/RGO and HRGO samples. Inset shows the zoomed area.

The FTIR spectrum shown in Fig. 2 indicates the presence of carboxyl, hydroxyl, and epoxy oxygen groups. The high intensity peaks in Ag/RGO spectrum is due to the interfacial interaction of AgNPs with the surface oxygen functionalities of RGO^30^. The FTIR spectrum of HRGO as shown in Fig. 2 shows that the intensities of the absorption bands, which can be ascribed to carboxyl, hydroxyl, and epoxy oxygen groups as in the FTIR spectrum of the Ag/RGO (see inset of Fig. 2), are reduced. This indicates that the oxygen containing groups have been effectively reduced and Ag has been removed during the formation of HRGO.

**Figure 3:**
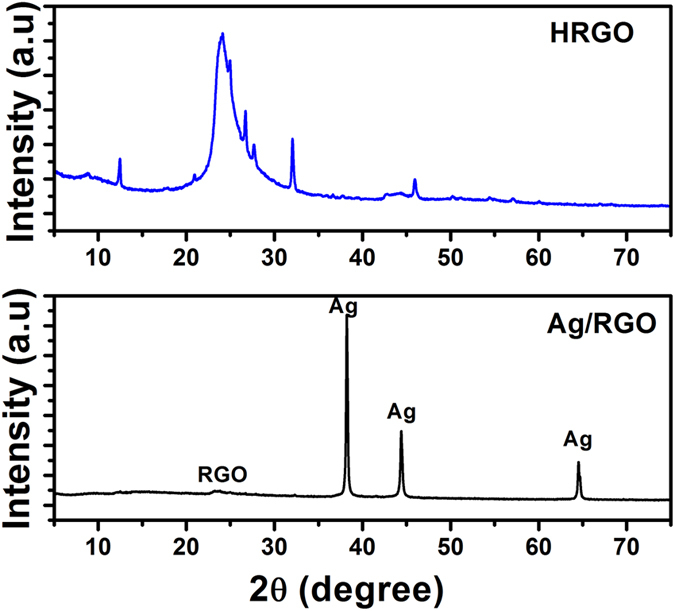
XRD patterns of Ag/RGO and HRGO samples.

Figure 3 shows the XRD pattern of the Ag/RGO and HRGO samples in which porous structure and the crystallinity of the HRGO can be further demonstrated. The XRD pattern of the Ag/RGO exhibits characteristic diffraction peaks of Ag and RGO, which is very well matched with the standard JCPDS of Ag^0^ (ICDD 04-0783). Intensity of Ag peaks is stronger than that of the RGO peaks which shows that Ag is reside successfully on the surface of the RGO sheets. Interestingly, the XRD pattern of the HRGO exhibits weak (001) reflection, indicating well-aligned sheet-to-sheet structures. As shown in Fig. 3, the XRD pattern of the HRGO exhibits a strong diffraction peak at diffraction angle of ~25.6 degree and other weak peaks correspond to HRGO, suggesting a porous structure of HRGO formed by the stacking of the sheets^31^,^32^. No peak related to Ag was observed in the XRD pattern of HRGO, indicating that Ag was successfully removed and created holes. The broad peak width indicates the non-uniformity of these holes, which is consistent with the microstructures of the HRGO building blocks. Despite the holey properties of the starting graphene sample, such as electron mobility, electrical conductivity, thermal conductivity, and mechanical properties, should be largely preserved. It is these properties of graphene that make it such an attractive material, so the fact that the HRGO samples retain them is very important for their subsequent applications that are dependent upon these properties^33^.

**Figure 4:**
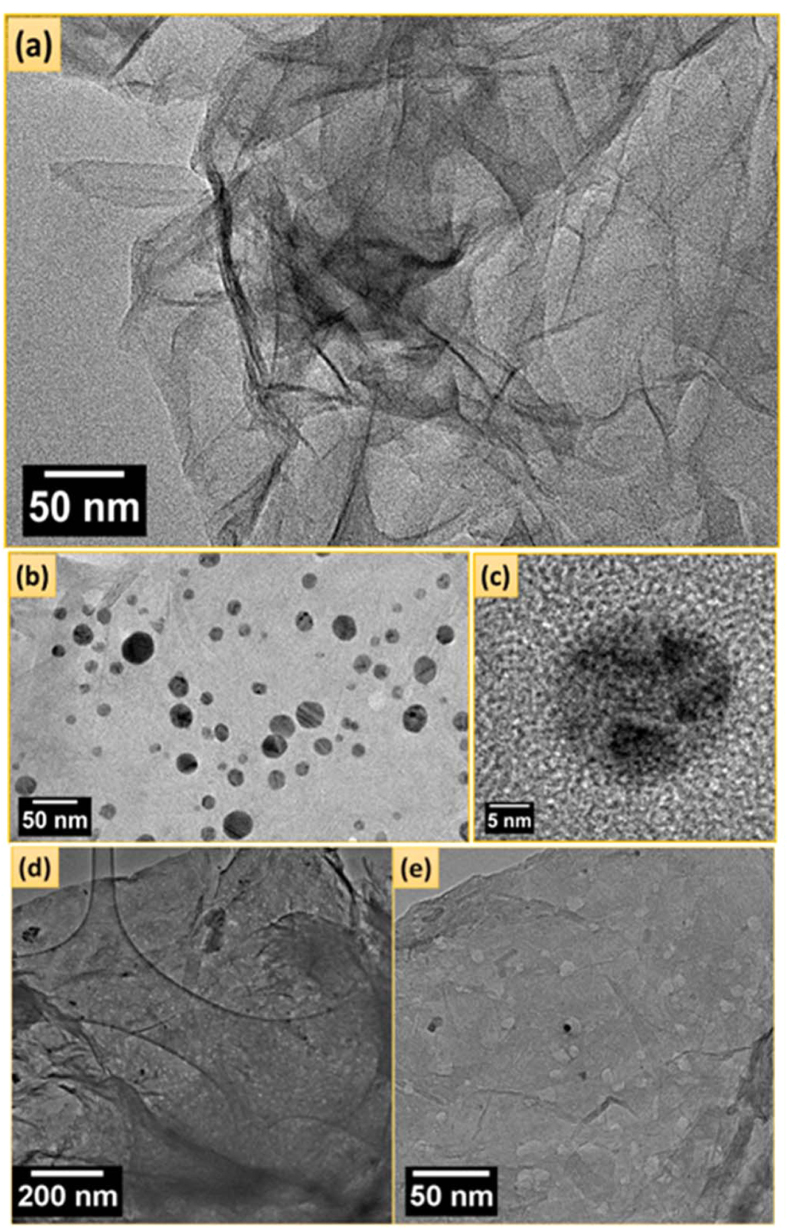
TEM images of (**a**) RGO, (**b**) Ag/RGO, (**c**) single Ag nanoparticles on RGO sheet, (**d**) HRGO at low magnification, (**e**) HRGO at high magnification.

Figure 4(a) depicts the TEM image of RGO sheets, in which clear sheet like structure is visible. Figure 4b show the TEM images of Ag/RGO revealing a sheet like structure on which Ag nanoparticles can be clearly seen. The metallic Ag nanoparticles were well distributed on the RGO surface (see Fig. 4b) and single Ag nanoparticle on RGO sheet is shown in Fig. 4c. Figure 4d shows a TEM image of the HRGO obtained from Ag nanoparticles on RGO sheets via the microwave reaction, followed by refluxing with nitric acid. The holey structure of the graphene sheets can be clearly visualized where RGO sheets consists of holes in the basal planes of graphene. No Ag nanoparticles were visually found by microscopic analysis of the final product of HRGO. In addition, the lack of Ag signals for these samples analyzed using both XPS and XRD confirmed the complete removal of the metal. After nitric acid treatment of Ag/RGO sample in microwave, a significant amount of holes appeared on the surface of RGO sheets as shown in Fig. 4d. Most holes were associated with at least one Ag nanoparticle which is clear in Fig. 4e. Some holes also appeared as tracks, which were apparently associated with directional motion of the attached Ag nanoparticles. Nevertheless, Ag nanoparticles with larger sizes typically yielded holes of larger diameters (or tracks of larger widths). Each hole or track might not have originated from only one Ag nanoparticle since those from multiple adjacent Ag nanoparticles could merge to form branched tracks or one single hole. Therefore, the above observations clearly indicate that Ag nanoparticles catalysed the oxidation of the graphene carbon atoms that were in contact with them, while the carbons that were not associated with the noble metal nanoparticles remained unaffected in the graphene structure. The holes in these samples penetrated the entire thicknesses of most HRGO nanosheets. The underlying stacks of HRGO sheets were clearly visible through these holes and tracks, which suggest better access for the electrolyte into the entire HRGO structure and hence better lithium-ion transport and intercalation mechanisms. Therefore, the electrolyte can penetrate deep into the HRGO anode not only via the holes and tracks on the film surface but also along the in-plane direction through the large continuous channels that are formed between the expanded HRGO sheets. This could play an important role in facilitating lithium-ion diffusion and intercalation, particularly at high charge/discharge rates.

**Figure 5:**
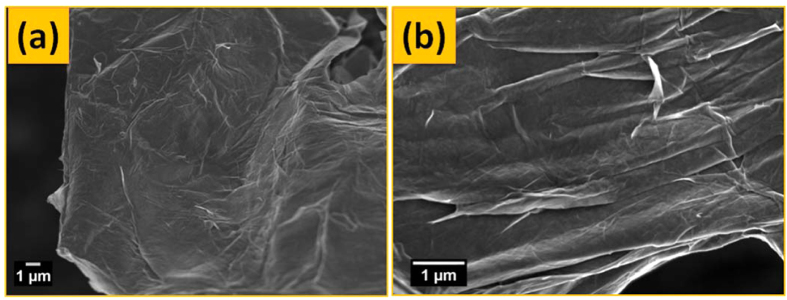
(**a**) Low, and (**b**) high magnification FESEM images of RGO without Ag.

To study the key role of Ag in the preparation of HRGO, the product RGO was prepared in similar process but without Ag. Figure 5 shows the FESEM images of RGO without Ag in which only nanosheets were observed and there is no existence of holes in the sheets of RGO. Thus, it might be reasonable to state that Ag played a catalytic role in the oxidation of graphene carbon atoms and created holes which resulted to HRGO.

**Figure 6:**
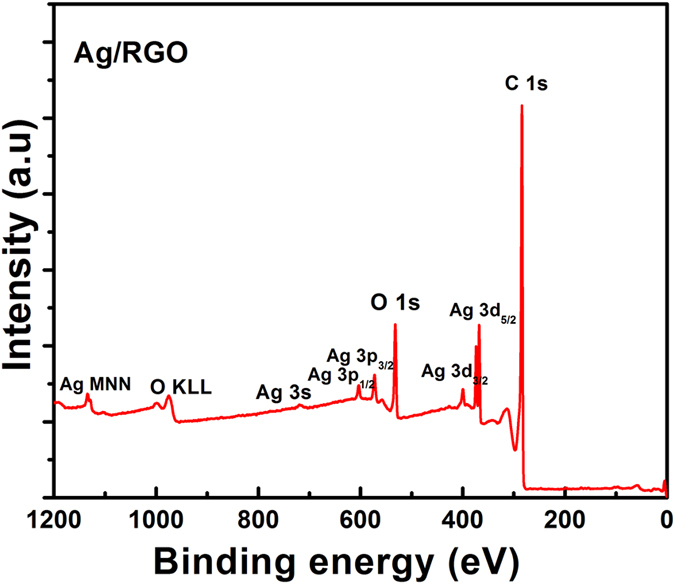
XPS spectrum of Ag/RGO sample.

**Figure 7:**
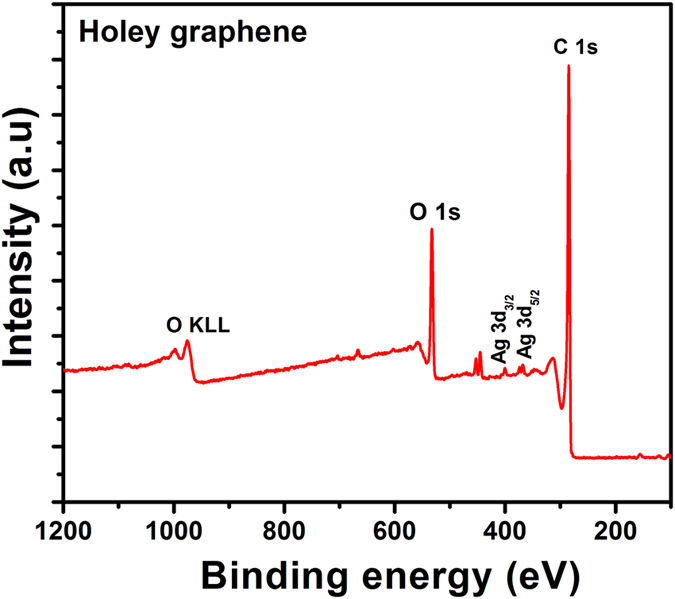
XPS spectrum of HRGO sample.

X-ray photoelectron spectroscopy (XPS) was used to study the surface chemistry of the Ag/RGO and HRGO samples. The survey spectra from X-ray photoelectron spectroscopy (XPS) as shown in Fig. 6 exhibit sharp Ag 3d_3/2_ and 3d_5/2_ peaks at 374.3 and 368.2 eV (Fig. S1(c)), also consistent with the presence of metallic Ag in Ag/RGO sample. While, the results shown in the survey spectrum of HRGO in Fig. 7 confirm that the majority of oxygen moieties are expelled in HRGO, and the lack/weakening of Ag signals for these samples confirmed the removal of the Ag metal. Formation of HRGO is evidenced by the dominant C-C sp2 peak^34^ at 284.2 eV in C1s and less intense oxygen peak in o1s spectra as shown in Figs 7 and S2(a,b), respectively. Presence of oxygen-containing functional groups has been shown to promote irreversible capacities in graphene-based electrodes^35^, and hence, a lower oxygen concentration is desirable for improved reversible capacities and high coulombic efficiencies.

Microwave assisted deposition of Ag nanoparticles onto RGO sheets and its oxidation in furnace was investigated using TGA analysis. For consistent comparison, the heating rate was kept at 5 °C/min and the air flow rate was at 50 ml/min. After deposition of Ag nanoparticles, the weight loss threshold significantly occurred at ~260 °C and shown in Fig. S3, which was reduced to 250 °C after the formation of HRGO. These results indicated that Ag nanoparticles acted as catalysts and reduced the activation energy of graphene oxidation so that it could occur at a much lower temperature.

**Figure 8:**
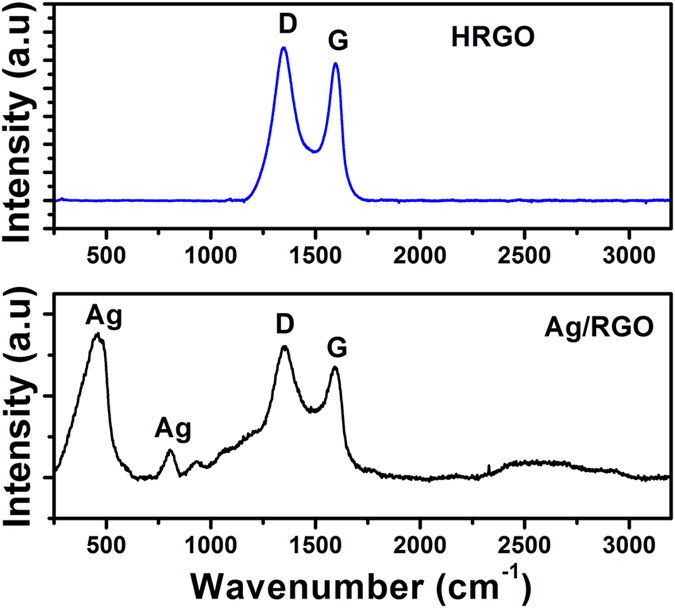
Room temperature Raman spectra of Ag/RGO and HRGO.

Figure 8 shows the Raman spectra of the Ag/RGO and HRGO samples. Sufficient information about the structure of graphene can be extracted from the Raman spectrum. The peaks at 1347 cm^−1^ and 1601 cm^−1^ were attributed to sp^3^ (D band) and sp^2^ (G band) hybridization carbon atoms, respectively are shown in figure for both the samples. However, peaks at 400 and 950 cm^−1^ were ascribed to the modes in Ag^36^ are visible in the spectrum of Ag/RGO only. It is well known that the D band is a measurement of “defects” or disruption of sp^2^ bonds of the carbon and sp^3^ formation^36^, while the G band is the result of the first-order scattering of the E_2_g mode of sp^2^ carbon domains. Both the position and intensity of D and G bands are highly susceptible to the structural changes of the carbon matrix, and there are many factors which can affect the position and intensity of D and G bands, such as doping, layer numbers, defects, strains, substrates, etc.^36^. The ratio of intensity of D/G bands is a measure of the defects present on graphene structure. The D/G ratio of HRGO was found to be 1.19, higher than that of D/G ratio of 1.10 of Ag/RGO. The absence of Ag signals and increase in D/G ratio in the Raman spectrum of HRGO sample clearly indicates the successful formation of defects in the form of holes in graphene resulted to HRGO.

**Figure 9:**
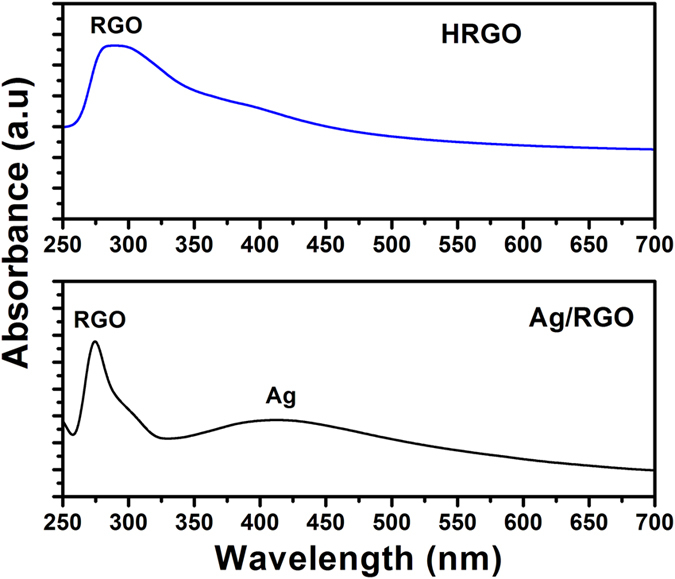
UV-Vis absorption plot of Ag/RGO and HRGO samples.

UV-Vis spectroscopy experiment was done to validate the optical properties of Ag/RGO and HRGO samples. Figure 9 shows UV-Vis spectra of Ag/RGO and HRGO product. It is clear from the spectrum of Ag/RGO that the peaks at ~272 nm is due to RGO and the peak at ~422 nm belongs to Ag nanoparticles, respectively. Whereas, in the HRGO spectrum, there is only one clear peak at ~270 and no peak related to Ag was observed which indicated that Ag was successfully removed from the RGO surface.

**Figure 10:**
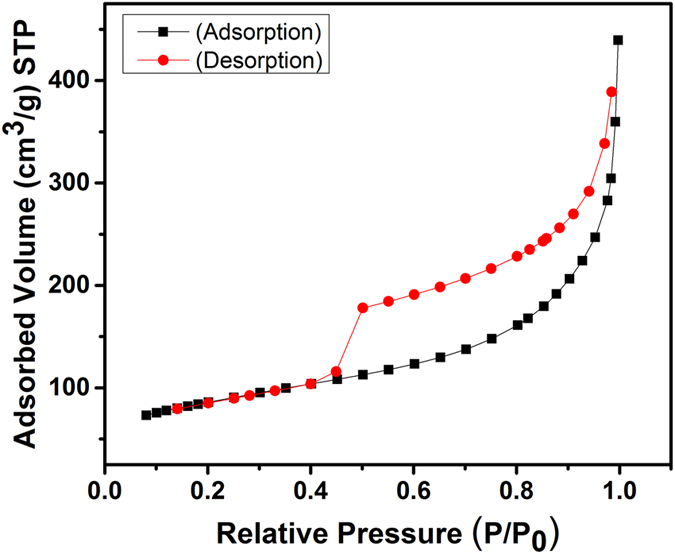
Nitrogen adsorption/desorption isotherms of the HRGO.

**Figure 11:**
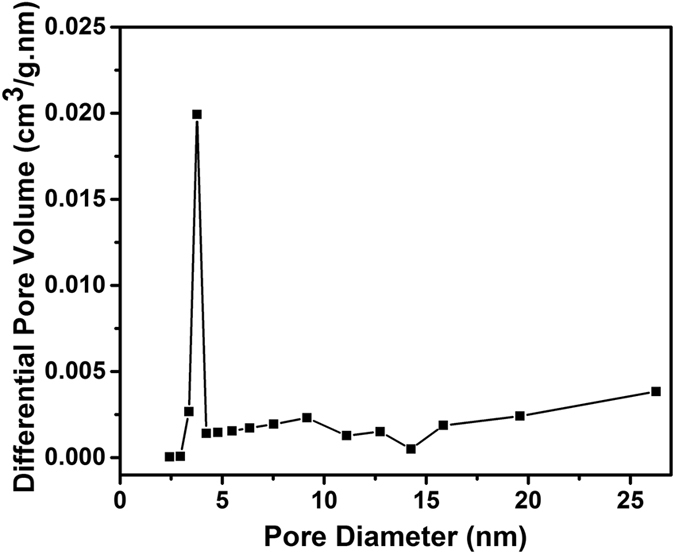
BJH pore size distribution plot of HRGO.

The porous nature of the HRGO was further confirmed by nitrogen physisorption measurements. Their adsorption–desorption isotherms exhibit a typical IV hysteresis loop (Fig. 10), which is characteristic of mesopores with different pore sizes. Barrett–Joyner–Halenda (BJH) calculations disclose that the pore size distribution is in the range of 2–5 nm (Fig. 11). A high BET surface area of 457 m^2^/g is observed for the HRGO, which is significantly higher than that of natural graphite (8.5 m^2^/g), pristine graphene (275 m^2^/g), and the pure holey graphene foam (131 m^2^/g) reported in the literature^37^,^38^,^39^. Because the BET surface area mainly originated from the mesopores and micropores, we further conducted a methylene blue (MB) adsorption investigation to evaluate the macroporous surface area of the HRGO. According to the surface area measurement using methylene blue method, the HRGO has a specific surface area of 945 m^2^/g, which is higher than that of the surface area obtained from BET analysis.

The adsorption of MB has a long history of use as a method of surface area determination^40^; it has been adopted widely for solids of a variable nature (oxides, graphite, yeast, activate carbons, etc.)^41^. It was found that there were relatively large differences between the surface area values obtained by solution Adsorption (S_MB_) and by nitrogen adsorption (S_BET_). The surface are obtained by MB adsorption method is usually higher than that of the nitrogen adsorption BET method. The surface area of HRGO obtained by MB adsorption method was found to be higher than that obtained by nitrogen adsorption BET method. This can be attributed to the swelling property of HRGO. In liquids, HRGO sheets can be separated by penetration of liquid molecules between sheets. This leads to the characteristic swelling of HRGO in certain liquids, particularly in water. Because the individual sheets within the crystal can be separated in the HRGO, not only the external area, but also internal surface area is available. Owing to the penetration of MB cations in aqueous solutions between the sheets of the HRGO, the surface area of HRGO was found to be higher than that of by BET method. The existence of the holes in the HRGO can facilitate the transport of lithium ions in HRGO, allowing a better access of the graphene structure to the electrolyte. This along with the large surface area, which increases the active sites for the lithium ion intercalation, makes us believe that the obtained HRGO can exhibit improved performance as an anode in LIBs.

**Figure 12:**
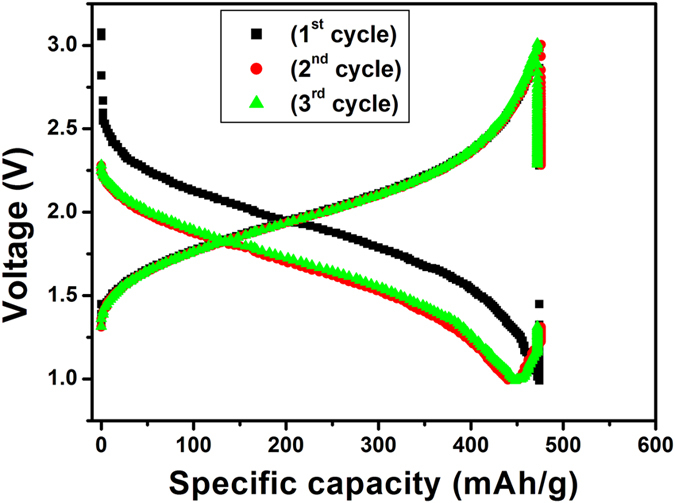
Initial galvanostatic charge/discharge curves for HRGO obtained at a current density of 100 mA/g.

**Figure 13:**
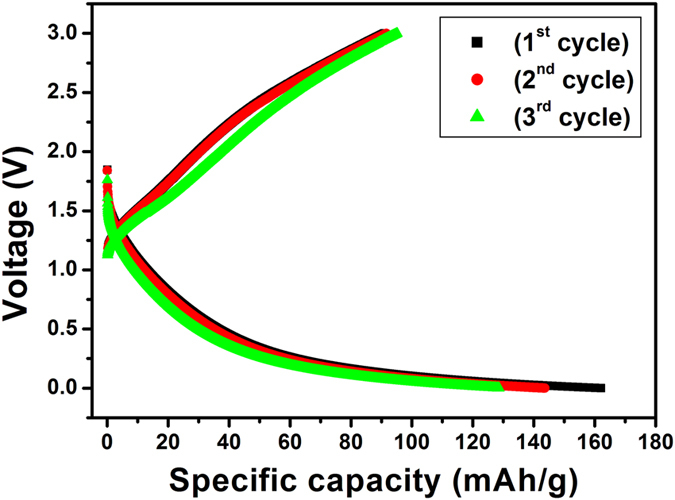
Initial galvanostatic charge/discharge curves for RGO without holes obtained at a current density of 100 mA/g.

In order to evaluate the performance of HRGO sheets, we investigated electrochemical response in terms of Li intercalation and deintercalation. In this work, for the sake of clarity, a typical charge/discharge cycle is defined as (i) a charge cycle; when Li insertion occurs until a lower limit voltage (0.005 V) is reached (ii) a discharge cycle; when Li extraction occurs until a cut-off voltage (3 V) is reached. Figure 12 shows the 1st, 2nd, and 3rd galvanostatic charge/discharge curves of HRGO sample from 0.005 to 3.0 V at a current density of 100 mA/g. The voltage profiles in Fig. 12 confirm the intercalation of the Li ions into the electrode structure. During first charge cycle, a voltage plateau at around 0.9 V is observed which is briefly maintained until the voltage drops steadily to 0.005 V. The discharge capacity during first cycle is observed at a high value of 423 mAh/g. The irreversible capacity loss observed during the first cycle is mainly attributed to the formation of a solid electrolyte interphase (SEI) layer on the surface of the HRGO electrode and/or the reaction of lithium ions with the residual oxygen-containing functional groups^42^. To confirm the role of the holes in the HRGO can facilitate the transport of lithium ions in HRGO, charge/discharge analysis of RGO without holes was performed and compared with the HRGO. It is clear from galvanostatic charge/discharge curves of RGO without holes as shown in Fig. 13 that the discharge capacity of 164 mAh/g for the 1^st^ cycle was observed, which is ~2.5 fold less than the discharge capacity of HRGO. This indicates that the holes in HRGO facilitate the transport of lithium ions in HRGO, allowing a better access of the graphene structure to the electrolyte.

**Figure 14:**
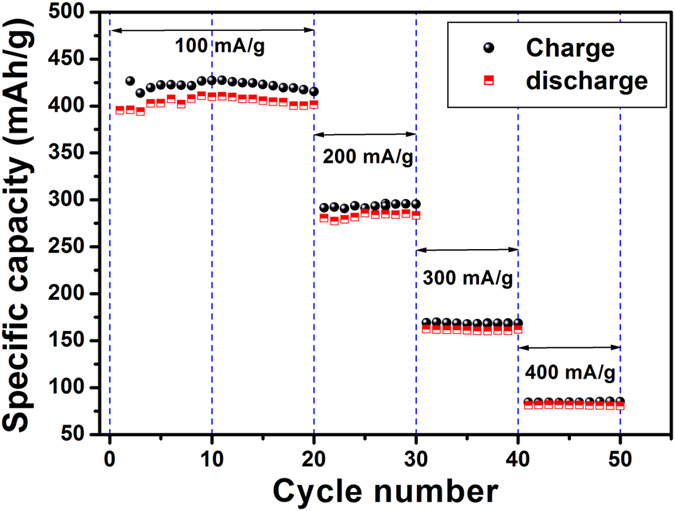
Rate capabilities of the HRGO at various current densities.

In order to further examine the behavior of HRGO at different current densities, rate capability test has been conducted as shown in Fig. 14. Both charge/discharge capacities have been evaluated at various current densities ranging from 100 to 400 mA/g. Initially, degradation in the value of cell capacity is observed at 100 mA/g, but stabilizes at a relatively constant value once the material has gone through repeated cycles. At 200 mA/g, the material capacity is 293 mAh/g, while at high current density of 400 mA/g, the capacity was decreased to 85 mAh/g. During the test, the material behavior is very stable with no abnormal drift even at high current densities; in fact, by reducing the current density back to 100 mA/g the cell capacity is completely restored.

**Figure 15:**
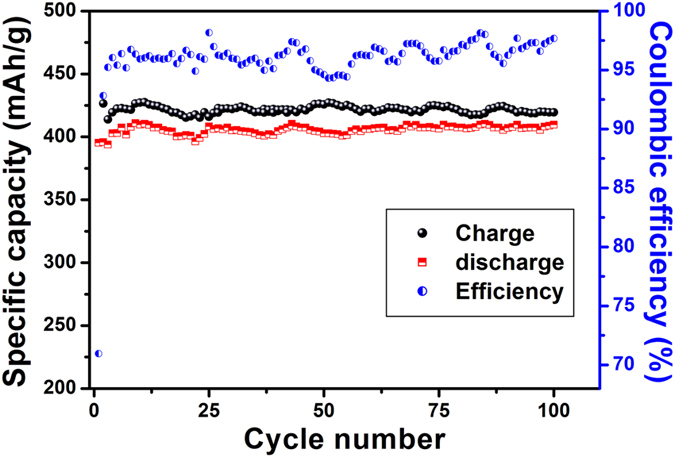
Cyclic performance and Columbic efficiency obtained at a current density of 100 mA/g for HRGO.

**Figure 16:**
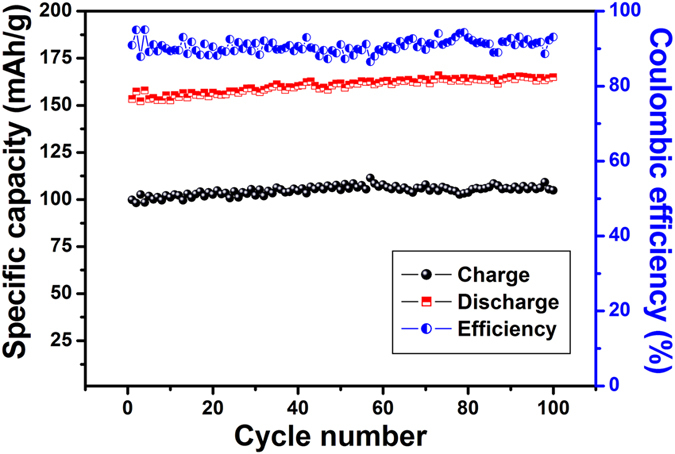
Cyclic performance and Columbic efficiency obtained at a current density of 100 mA/g for RGO without holes.

The results of cyclic performance test are also quite remarkable as shown in Fig. 15. At a current density of 100 mA/g a high capacity of 400 mAh/g is retained for 100 cycles. Also, the coulombic efficiency is around 98% showing excellent reversible charge–discharge behaviour. The cyclic performance test for RGO without holes was also performed and compared with the HRGO. Figure 16 shows that at a current density of 100 mA/g, a capacity of 152 mAh/g is retained for 100 cycles, which is lower than that of the capacity of HRGO.

Compared to the theoretical discharge capacity (372 mAh/g, corresponding to the adsorption of the Li + ions on one side of the graphene nanosheet), and RGO without holes, the discharge capacity shown here by the HRGO is significantly high. It is worth mentioning here that this superior performance of larger surface area HRGO electrode is due to the holes, which provides better access for the electrolyte into the entire HRGO structure and hence better lithium-ion transport and intercalation mechanisms.

**Figure 17:**
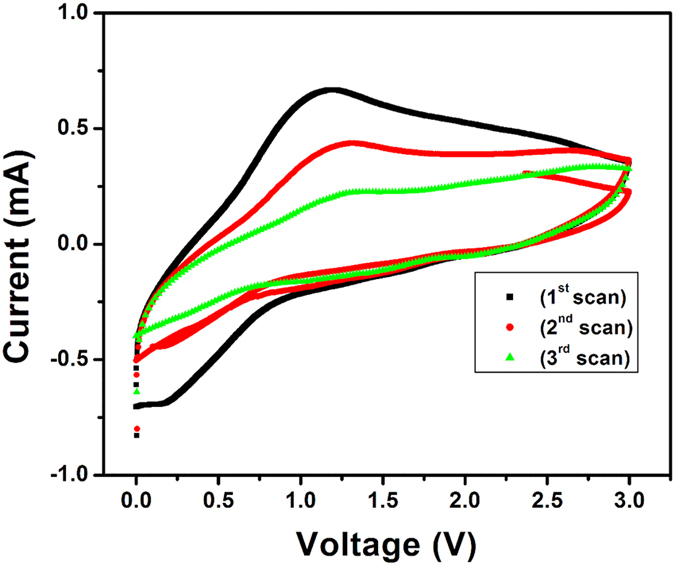
Cyclic voltammograms of HRGO electrode in 1 M solution of LiPF_6_ in 1:1 (v/v) mixture of ethylene carbonate (EC) and dimethyl carbonate (DMC) electrolyte with the Li as the counter and reference electrode at a scan rate of 5 mV/s.

**Figure 18:**
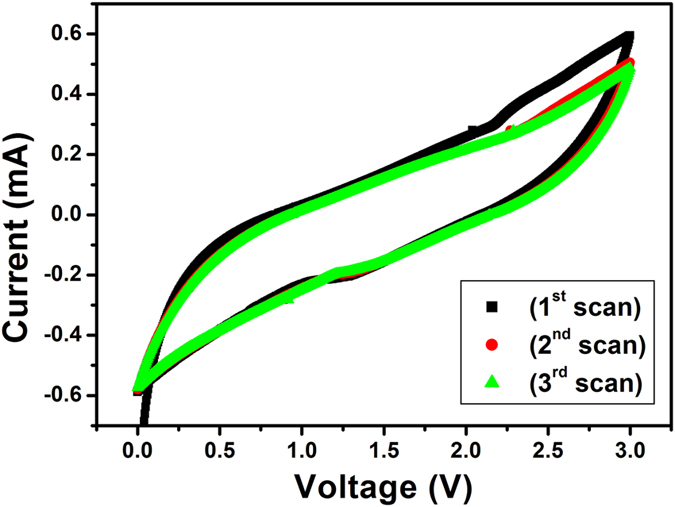
Cyclic voltammograms of RGO without holes electrode in 1 M solution of LiPF_6_ in 1:1 (v/v) mixture of ethylene carbonate (EC) and dimethyl carbonate (DMC) electrolyte with the Li as the counter and reference electrode at a scan rate of 5 mV/s.

The cyclic voltammograms (CVs) of the HRGO are shown in Fig. 17. The CV curves in Fig. 17 exhibit a typical electrochemical characteristic of graphite, showing that the Li^+^ions can reversibly insert/extract into/from the HRGO. During the first cathodic scan, peak at ~0.8 V is observed, which is indicative of single-phase Li insertion^43^. In the subsequent anodic scan, a single peak at 1.15 V is observed. During the 2^nd^, and 3^rd^ scans of the CV analysis, well-defined cathodic and anodic peaks are observed which are maintained at constant redox potentials. However, a decrease in the current peak intensity is noticed in comparison to the 1st scan, indicative of the irreversible capacity. This is in agreement with the charge/discharge cycles shown in Fig. 12. For a comparative study, CVs of RGO without holes was also performed and shown in Fig. 18. During the first, 2^nd^, and 3^rd^ cathodic and anodic scan, characteristics cathodic and anodic peaks were observed. However, the intensity of current peak is much lower as compared with the HRGO, which indicates that HRGO has superior voltage characteristics.

**Figure 19:**
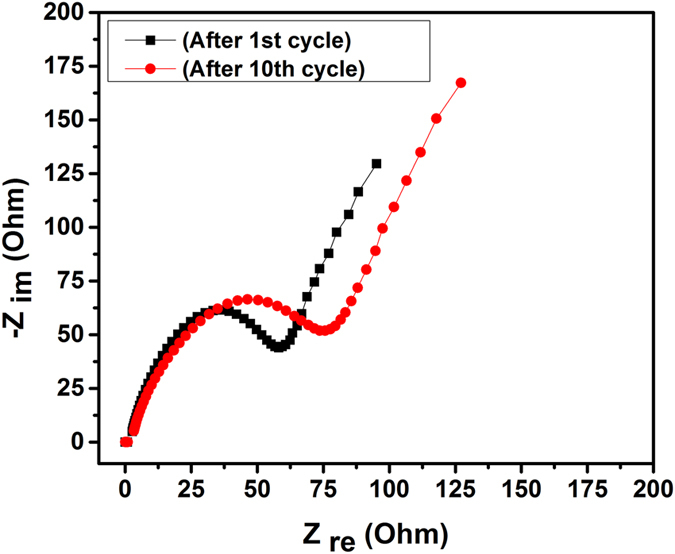
Electrochemical impedance spectra of HRGO from 100 kHz to 1 Hz in the form of Nyquist plots obtained for cells after 1^st^ cycle and 10^th^ cycle.

**Figure 20:**
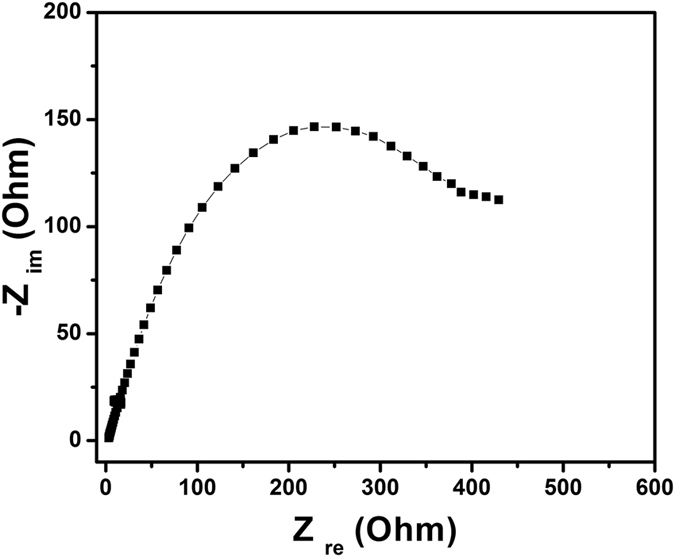
Electrochemical impedance spectra of RGO without holes from 100 kHz to 1 Hz in the form of Nyquist plots obtained for cells after 1^st^ cycle.

For further information regarding the factors responsible for the Li^+^ transfer rate into the HRGO, EIS measurements were carried out during the various stages of the cycling. Figure 19 shows the comparison between the impedance spectra in the form of Nyquist plots for the cells after 1^st^ and 10^th^ cycles. Each Nyquist plot is composed of a semicircle in the high-medium frequency region, and a linear tail in the low frequency region, which are ascribed to the charge transfer and mass transfer of Li^+^ions, respectively^44^. The diameter of the semicircle for the 1^st^ cycle corresponding to a resistance of 37.4 ohm. However, for the 10^th^ cycle, the spectra show a significant increase in the diameter of the semicircle which resulted to the increase in the charge transfer resistance to 57.1 ohm. The charge transfer resistance of the HRGO after 1^st^ cycle is only 37.4 ohms, prominently lower than those reported for graphene-based materials (40–70 ohms), indicating the charger transfer and mass transfer of lithium ions was much more conductive. To see the effect of holes on charge transfer, EIS measurements of RGO without holes was performed and charge transfer resistance of both the RGO without holes and HRGO compared. It was observed from nyquist plot of RGO without holes as shown in Fig. 20 that charge transfer resistance is much higher than that of HRGO, which indicates that holes might play key role in the enhanced electrochemical performance. The low charge-transfer resistance of the HRGO can be attributed to its porous structure, which allows improved diffusivity of Li ions in the electrode and a better access for the electrolyte into the HRGO. These results showed that the enhanced electrochemical performance of the HRGO demonstrated above is due to the combined result of its improved Li-ion diffusivity and decreased charge-transfer resistance. The porous structure of HRGO can greatly reduce the effective diffusion distance for the Li ions and buffer against the local volume change during Li insertion–extraction, resulting in greatly improved rate capability and cycling stability. This along with its large surface area, which provides more active sites for Li insertion, greatly increases the performance of the HRGO anode.

## Conclusions

A novel microwave irradiation method combined with Ag nanoparticles deposition on the RGO sheets and then acid treatment procedure has been used for the fabrication of HRGO. XRD, Raman, UV, XPS and morphological characterization confirmed the successful removal of Ag nanoparticles in Ag/RGO product, which then finally resulted to unique HRGO structure. The random stacking of the HRGO leads to the formation of a 3-dimensional porous solid, which can be used as a superior anode material for LIBs. A good electrochemical response in terms of charge/discharge capacity, rate capability, cyclic performance, and columbic efficiency was observed. This excellent performance is because of the holey structure of HRGO which provide more active sites for Li insertion, high electrical conductivity, low charge-transfer resistance across the electrolyte– electrode interface, reduced effective diffusion distance for the Li ions, and improved structural stability against the local volume change during Li insertion–extraction. In near future, this research will open up a new direction on the potential applications of HRGO with their conductive nature of and their porous structure to be used as advanced electrode materials in energy storage applications.

## Additional Information

**How to cite this article**: Alsharaeh, E. *et al*. Novel synthesis of holey reduced graphene oxide (HRGO) by microwave irradiation method for anode in lithium-ion batteries. *Sci. Rep.* **6**, 29854; doi: 10.1038/srep29854 (2016).

## Supplementary Material

Supplementary Information
