# Supplementary material for: Novel synthesis of holey reduced graphene oxide (HRGO) by microwave irradiation method for anode in lithium-ion batteries
Source: Sci Rep. 2016 Jul 26;6:29854. doi: 10.1038/srep29854 (PMC4960613; doi:10.1038/srep29854)
Supplement: Supplementary Information [file srep29854-s1.pdf]

Novel synthesis of holey reduced graphene oxide (HRGO) by microwave irradiation method for anode in lithium-ion batteries

Edreese Alsharaeh[a], Faheem Ahmed[a], Yazeed Aldawsari[a,] and Majdi Khasawneh[a], Hatem Abuhimd[b], Mohammad Alshahrani[b]

[a]*College of Science and General Studies, Alfaisal University, P.O. Box 50927, Riyadh, 11533, Saudi Arabia*

[b]*National Nanotechnology Center, King Abdulaziz City for Science and Technology*

P.O Box 6086, Riyadh 11442, Saudi Arabia

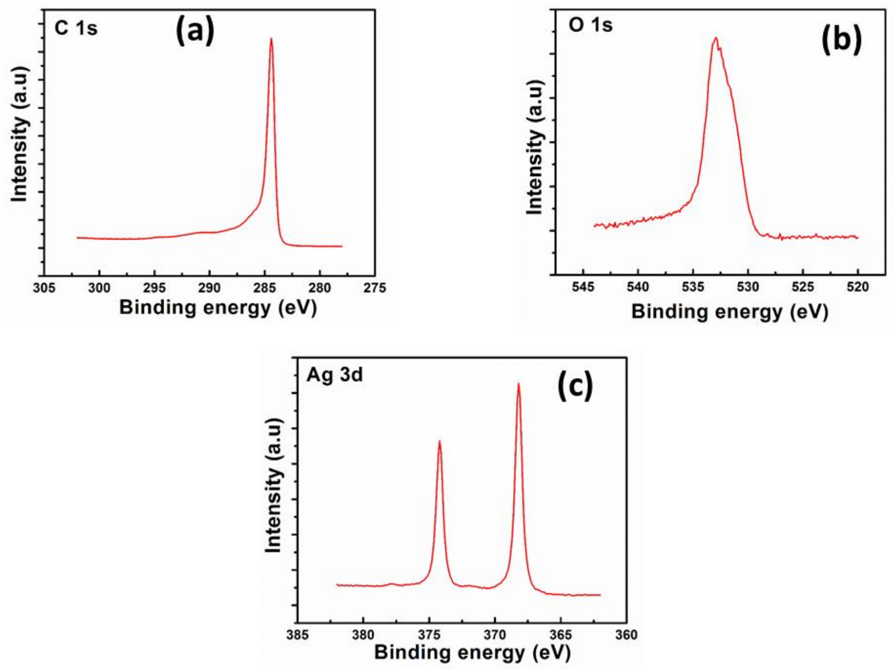

Fig. S1: XPS spectra of Ag/RGO (a) C1s, (b) O1s, (d) Ag3d.

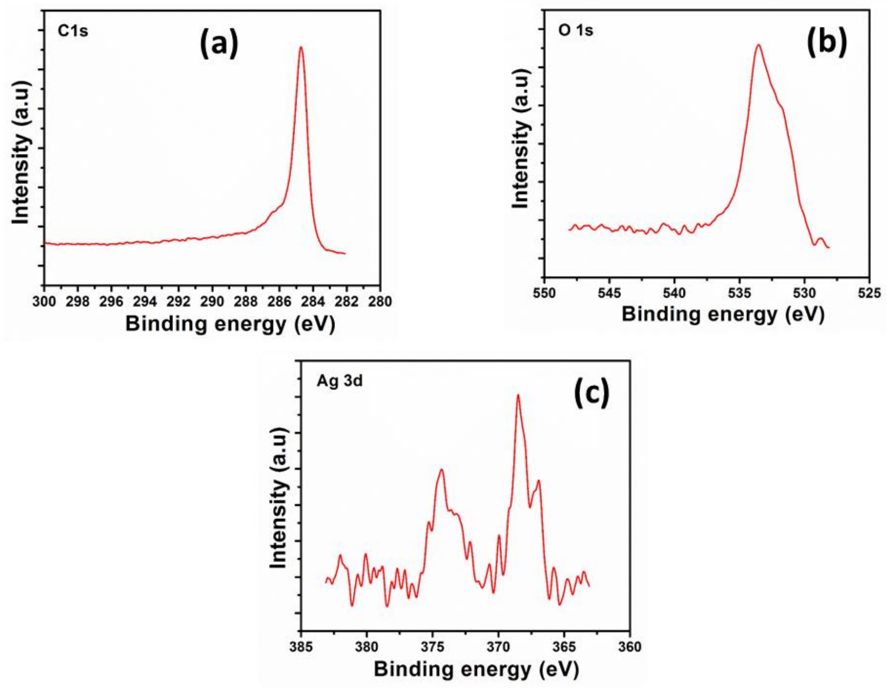

Fig.S2: XPS spectra of HRGO (a) C1s, (b) O1s, (d) Ag3d.

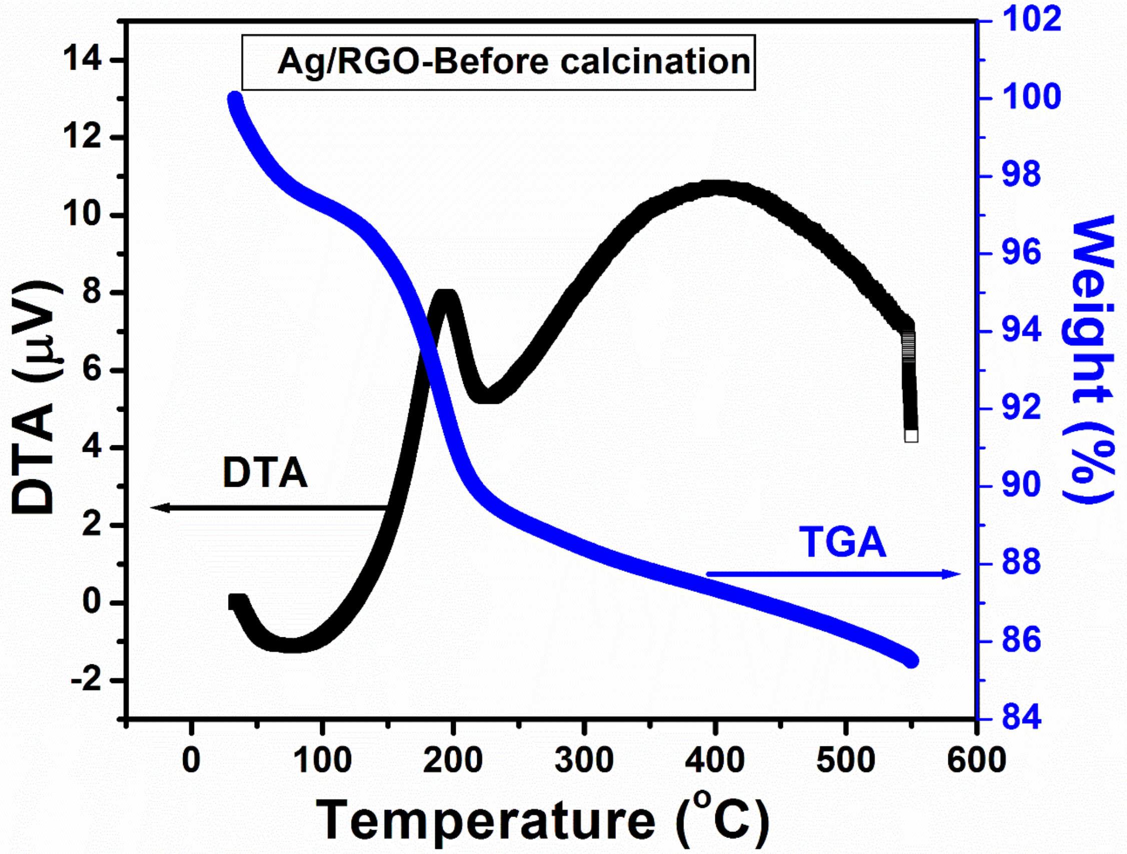

Fig.S3: TGA/DTA plot of Ag/RGO sample.
